# Effectiveness of Thrombolytic Therapy in Acute Embolic Stroke due to Infective Endocarditis

**DOI:** 10.4061/2010/841797

**Published:** 2009-11-09

**Authors:** Siva P. Sontineni, Aryan N. Mooss, Venkata G. Andukuri, Susan Marie Schima, Dennis Esterbrooks

**Affiliations:** Division of Cardiology, Creighton University, Omaha, NE 68131, USA

## Abstract

*Objective*. To identify the role of thrombolytic therapy in acute embolic stroke due to infective endocarditis. *Design*. Case report. *Setting*. University hospital. *Patient*. A 70-year-old male presented with acute onset aphasia and hemiparesis due to infective endocarditis. His head computerized tomographic scan revealed left parietal sulcal effacement. He was given intravenous tissue plasminogen activator with significant resolution of the neurologic deficits without complications. *Main Outcome Measures*. Physical examination, National Institute of Health Stroke Scale, radiologic examination results. *Conclusions*. Thrombolytic therapy in selected cases of stroke due to infective endocarditis manifesting as major neurologic deficits can be considered as an option after careful consideration of risks and benefits. The basis for such favorable response rests in the presence of fibrin as a major constituent of the vegetation. The risk of precipitating hemorrhage with thrombolytic therapy especially with large infarcts and mycotic aneurysms should be weighed against the benefits of averting a major neurologic deficit.

## 1. Introduction

The safety and efficacy of thrombolytic treatment of acute ischemic stroke due to septic brain embolism is not well established [1]. Large trials of thrombolytic therapy of acute ischemic stroke have excluded patients with septic embolization. Published systematic reviews do not address the role of the thrombolytic therapy in the setting of septic embolization to the brain, such as in infective endocarditis [2]. 

Stroke is the most common of neurologic complications associated with infective endocarditis [3]. The treatment and prevention strategies of this complication are still in evolution. The prevention rests on initiating early antibiotic therapy and consideration of surgery for recurrent events. The role of thrombolytic therapy for acute ischemic strokes in addition to antiplatelet therapy for the prevention of recurrent strokes has increasingly been recognized. We present here our experience of thrombolytic treatment of acute ischemic stroke in a patient with unsuspected infective endocarditis and the available evidence in the literature.

## 2. Clinical Summary

**Figure 1:**
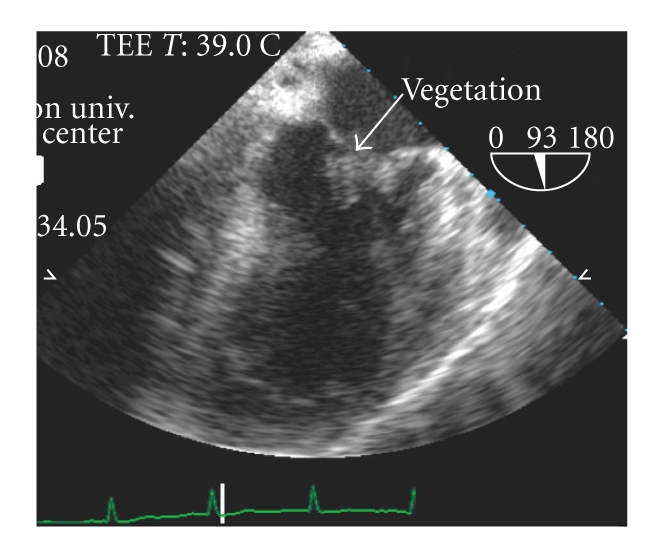
Transesophageal echocardiogram revealing vegetation on the mitral valve leaflets.

**Figure 2:**
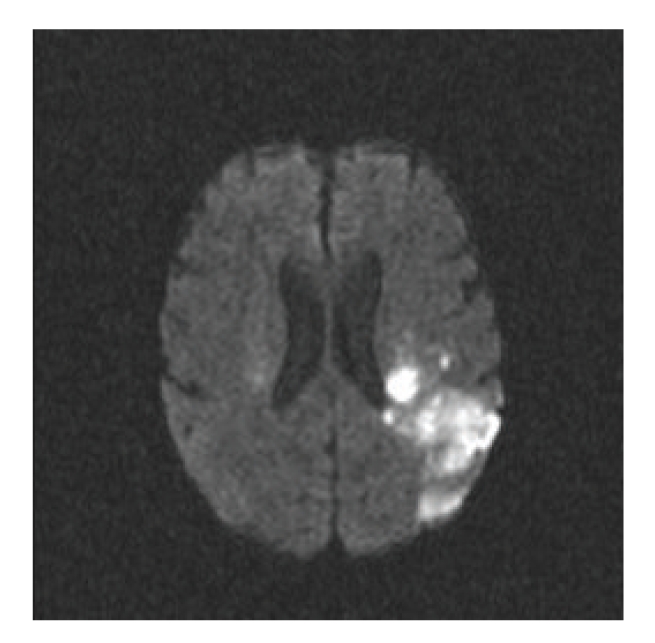
Diffusion weighted MRI after thrombolysis showing left parietal hyperintensities. Areas of focal intensities were also identified in other location including the right cerebral hemisphere, consistent with embolic source as a likely cause of these bilateral and sporadic hyperintense lesions.

A 70-year-old Caucasian male with history of hypertension and hyperlipidemia presented to a remote emergency room with altered mental status, right hemiparesis, and aphasia. On initial evaluation his National Institutes of Health Stroke Scale (NIHSS) score was 13. A computed tomography scan of the head revealed left parietal hypodense area without haemorrhage. Two and half-hour after the onset of symptoms, intravenous tissue plasminogen activator (t-PA) was started at the outlying emergency room and then transported to our University hospital for further management. On presentation, patient had already finished his t-PA infusion. On physical exam he had fever (39.7°C) and cardiac auscultation revealed a blowing 3/6-holosystolic murmur at the apex. A transthoracic echocardiogram revealed left ventricular ejection fraction of 60–65% with moderate mitral regurgitation. Transesophageal echocardiogram revealed 1.6 × 1.2 cm vegetation on the anterior mitral valve leaflet (Figure 1). Blood cultures returned positive for *Streptococcus pneumoniae* and he was started on penicillin G intravenous 5 × 10^6^ units every four hours. A diffusion weighted MRI of the brain (Figure 2) revealed bilateral acute ischemic areas (left parietal and frontal regions in the left middle cerebral artery distribution and in the right corona radiata and right occipital lobe) and magnetic resonance angiography was normal. Aspirin (81 mg) was started one day after thombolysis and penicillin G was continued for 6 weeks, followed by steady clinical improvement with the continued care of physical, occupational, and speech therapists. The NIHSS score was 5 at discharge with minimal residual aphasia. Two months later, he was evaluated for weight loss, anemia, and melena and was diagnosed with carcinoma of the colon for which he successfully underwent right hemicolectomy without any complication.

## 3. Discussion

The use thrombolytic therapy in patients with infective endocarditis suffering from embolic events is not well studied. Treatment with intravenous thrombolysis within 3 hours of symptom onset has become the standard of care in acute ischemic stroke [4]. Other treatment modalities such as intra-arterial thrombo lysis and catheter-based mechanical thrombus disruption therapies have been studied in the treatment of acute ischemic stroke with variable success [5–7]. The value and safety of these therapies in acute stroke caused by embolism related to infective endocarditis is evolving. Whether the increased risk of intracranial hemorrhage in patients with infective endocarditis is to be considered a contraindication for thrombolysis is unclear. Most of these strokes are ischemic and therefore may respond to thrombolysis. Our case example along with other reported cases of successful intravenous thrombolysis in patients meeting the standard criteria for intravenous t-PA could spur further studies to address this clinical scenario. Similar reports of successful intra-arterial thrombolysis of basilar artery and left internal carotid artery thrombosis in two subjects with infective endocarditis need further evaluation by well designed studies [1, 8]. We summarize the outcomes of these four cases in Table 1. Publication bias is a concern with such reports in the literature with only favourable outcomes being reported. 

Infective endocarditis is an endovascular microbial infection of cardiovascular structures, including large intrathoracic vessels and intracardiac foreign bodies. The commonly accepted pathogenetic theory is from endothelial injury with deposition of noninfective sterile thrombotic vegetations to transient bacteremia with microorganism adhesion (injury-thrombus-infection theory). The characteristic lesions consist of vegetations composed of platelets, fibrin, micro-organisms, and inflammatory cells [9]. Thus fibrinolytic therapy may be helpful in thrombus resolution by their action on the fibrin present in the vegetation itself or new fibrin that may develop after embolization. Local destructive effects, distant haematogenous seeding, immunologic and embolic phenomena can complicate the clinical course of infective endocarditis. 

Embolism occurs in 20–40% of infective endocarditis cases, but its incidence decreases to 9–21% after initiation of antibiotic treatment. The risk of embolism is especially high with large vegetations (>10 mm), mobile vegetations and during the early course of endocarditis therapy. The brain and spleen are the most frequent sites of embolism in infective endocarditis [10]. 

The overall incidence of stroke in patients with infective endocarditis is about 10% and can result either from embolic infarction or haemorrhage. Patients with mitral valve endocarditis are at a greater risk of stroke than patients with aortic valve endocarditis [3]. Cerebral embolism may manifest as a stroke of varying severity associated with fever, or may be asymptomatic [10]. In a retrospective analysis by Hart et al. [11], 74% of ischemic strokes were noted in native-valve endocarditis at presentation, 13% occurred within 48 hours after diagnosis, and 9% of infarcts are large. Hemorrhagic stroke in patients with infective endocarditis occurs during the early stages (60% within 48 hours) of clinical course and the mechanisms responsible can be septic arteritis, secondary hemorrhagic transformation of infarction, accompanying anticoagulation and mycotic aneurysms [12]. Hemorrhagic conversion of the ischemic infarct due to septic emboli is the most frequent mechanism followed by the rupture of pyogenic arteritis and mycotic aneurysms [13]. The presence of pathologic lesions such as pyogenic arteritis, mycotic aneurysms, and large infarcts due to cerebral embolism increases the risk of hemorrhage with thrombolytics in patients with infective endocarditis. The relatively low incidence of 6% of intracranial hemorrhage [11] and the fact that the fibrin thrombus constitutes most of the vegetation according to pathological studies [9] raise the question whether infective endocarditis should be a relative rather than an absolute contraindication in carefully selected patients who may potentially benefit from thrombolytic therapy, in the absence of mycotic aneurysm and large infarcts [8].

The cornerstone of medical treatment of infective endocarditis is to institute effective antibiotic therapy as soon as possible to reduce the mortality and morbidity from embolic complications and heart failure [10, 14]. The role of antiplatelet agents in the therapy of infective endocarditis has been increasingly recognized. Anavekar et al. [15] evaluated the effects of preceding antiplatelet therapy (aspirin, dipyridamole, clopidogrel, ticlopidine, or any of combination) on the risk of embolism in a cohort of 600 cases of infective endocarditis. In this retrospective study, embolic events occurred in 12.0% of patients who had received prior antiplatelet therapy compared to 28% patients who had not received therapy. This analysis demonstrates that the risk of symptomatic emboli associated with infective endocarditis is reduced in patients who received continuous daily antiplatelet therapy before onset of infective endocarditis.

Anticoagulation is still a matter of debate in infective endocarditis, since it can increase the risk of complications, mostly neurological. In a study of patients with native and prosthetic valve endocarditis, stroke was significantly more frequent among anticoagulated patients (20% versus 7%) than nonanticoagulated patients [16]. In a similar analysis, patients who were already on anticoagulation for prosthetic valves had the same embolic risk as compared to those who are not on anticoagulation [14]. Oral anticoagulation is ineffective in stroke prophylaxis, and their use may even be associated with an increased case fatality rate, especially for patients with *Staphylococcus aureus* infective endocarditis [17]. Thus the indications for anticoagulation in infective endocarditis remain similar to those in valvular heart disease, and anticoagulation with heparin should be maintained whenever a brain infarct is present, unless it is large and/or haemorrhagic [16]. European society of cardiology guidelines recommend to discontinue coumadin therapy and replace by heparin soon after the diagnosis of infective endocarditis [18].

In spite of these positive outcomes in these selected case reports, the mainstay of therapy of infective endocarditis remains prompt antibiotic therapy. In all cases of acute ischemic strokes, especially those with a history of fever, infective endocarditis need to be considered and promptly investigated with blood cultures and echocardiographic evaluation. Whenever the clinical suspicion is high for infective endocarditis, careful clinical judgement is required before instituting thrombolytic therapy, weighing the benefits of averting a major neurologic deficit with high NIHSS score versus precipitating cerebral hemorrhage. Prior imaging to identify the presence of mycotic aneurysm can be considered whenever feasible to guide the decision to some extent, however prior pathologic studies of infective endocarditis attributed only a minority of cerebral hemorrhages to the mycotic aneurysms [13]. Although current evidence is lacking for the effects of thrombolytic therapy on the clinical course of mycotic aneurysms, it is intuitive to consider increase the risk of cerebral hemorrhage with thrombolytic therapy in the presence of mycotic aneurysm. Additionally we believe publication bias in the literature on this subject is a significant issue with only favourable cases being reported. 

In conclusion, thrombolytic therapy in cases of stroke due to infective endocarditis manifesting with major neurologic deficits is a subject of controversy and needs careful evaluation in a randomized trial. The beneficial effects of therapy can be greatly jeopardised by the serious complications of intracranial hemorrhage. 

## Figures and Tables

**Table 1:** Clinical characteristics and outcomes of reported cases of thrombolysis in infective endocarditis.

Ref. no	Year	Age	Gender	IE suspected	Organism	Fever	Symptoms	Admission NIHSS Score	Imaging prior to thrombolysis	Thrombolytic used	Time interval	Microaneurysms	Discharge NIHSS score Bleeding complication
1	2003	31	F	Prethrombolysis	*Streptococcus mitis*	Absent	Limb weakness Vertigo Tinnitus Gaze palsies	13	CT scan: hypodensity of thalamus	Intra-arterial urokinase	5h	None on CT	NIHSS score 5, no hemorrhage

8	2009	12	F	Post thrombolysis	*Streptococcus pneumoniae*	Present	Acute hemiparesis Aphasia	18	MRI: multiple diffusion-restricted lesions; MRA absent flow in the left internal carotid artery	Intra-arterial t-PA	6h	None on MRI	NIHSS score 5, no hemorrhage

19	2007	56	M	Post thrombolysis	*Beta-hemolytic streptococcus*	Present	Acute hemiparesis Aphasia	15	CT scan: loss of insular ribbon with indistinctness of lentiform nuclei, no hypodensity	Intravenous t-PA	2h, 36 min	Unknown	NIHSS score 4, no hemorrhage

Our case	2009	70	M	Post thrombolysis	*Streptococcus pneumonia*	Present	Acute hemiparesis Aphasia	13	CT scan: hypodensity	Intravenous t-PA	2h, 30 min	None on MRA	NIHSS score 5, no hemorrhage

IE: infective endocarditis; M: male; F: female; NIHSS: National Institute of Health Stroke Scale; CT: computed tomography; MRI: magnetic resonance imaging; MRA: magnetic resonance angiography.
